# Lipid droplet dynamics in healthy and pyometra-affected canine endometrium

**DOI:** 10.1186/s12917-022-03321-5

**Published:** 2022-06-11

**Authors:** Natascha Leitner, Juraj Hlavaty, Susanne Heider, Reinhard Ertl, Cordula Gabriel, Ingrid Walter

**Affiliations:** 1 Institute of Morphology, Working Group Histology, University of Veterinary Medicine, Veterinaerplatz 1, A-1210 Vienna, Austria; 2 VetCORE Facility for Research, University of Veterinary Medicine, Veterinaerplatz 1, A-1210 Vienna, Austria

**Keywords:** Dog, Pyometra, Lipid droplets, PLINs

## Abstract

**Background:**

Accumulation of lipid droplets (LDs) was recently observed in pyometra-affected uteri. As data about their nature and function are missing we intended to compare the localization, quality and quantity of LDs in canine healthy and pyometra-affected tissues and in an in vitro model.

**Methods and results:**

We characterized LDs in healthy and pyometra uterine tissue samples as well as in canine endometrial epithelial cells (CEECs) in vitro by means of histochemistry, immunohistochemistry, transmission electron microscopy, western blot, and RT-qPCR. Oil Red O (ORO) staining and quantification as well as p-phenylenediamine staining showed a higher number of LDs in epithelial cells of pyometra samples. Immunohistochemistry revealed that the amount of LDs coated by perilipin2 (PLIN2) protein was also higher in pyometra samples. Transmission electron microscopy showed an increase of LD size in surface and glandular epithelial cells of pyometra samples. In cell culture experiments with CEECs, supplementation with oleic acid alone or in combination with cholesterol lead to an increased LD accumulation. The expression of PLIN2 at protein and mRNA level was also higher upon oleic acid supplementation. Most LDs were double positive for ORO and PLIN2. However, ORO positive LDs lacking PLIN2 coating or LDs positive for PLIN2 but containing a lipid class not detectable by ORO staining were identified.

**Conclusions:**

We found differences in the healthy and pyometra-affected endometrium with respect to LDs size. Moreover, several kinds of LDs seem to be present in the canine endometrium. In vitro studies with CEECs could show their responsiveness to external lipids. Since epithelial cells reacted only to oleic acid stimulation, we assume that the cyclic lipid accumulation in the canine endometrium is based mainly on triglycerides and might serve as energy provision for the developing early embryo. Further studies are necessary to verify the complex role of lipids in the healthy and pyometra-affected canine endometrium.

**Supplementary Information:**

The online version contains supplementary material available at 10.1186/s12917-022-03321-5.

## Background

The canine endometrium undergoes massive characteristic morphological changes during each sexual cycle. The remodeling includes the connective tissue of the endometrial stroma and also the surface, crypt, and glandular epithelium. One astonishing phenomenon is the accumulation of lipid droplets (LDs) in the surface and crypt epithelium of the canine endometrium during the luteal phase of the sexual cycle. This aggregation of LDs in the canine endometrium has been reported before [1–3]; however, the functional background for their explicit presence in the luteal phase as well as the kind of lipid within the LDs or their membrane coating proteins remain unclear. Endometrial lipid accumulation has been observed long ago in other species such as cows [4], ewes [5], or rats [6]. In cows, ewes and rats also the luteal phase was the main time of LD accumulation. In this context, the suggestion of Brinsfield and Hawk [7] and Wordinger et al. [4] that progesterone is the main hormone controlling lipid accumulation in the endometrium is reasonable.

In the pyometra affected uterus, a characteristic lipid storage pattern has been observed also in the surface and crypt epithelium [3]. Pyometra, a uterine disease associated with bacterial infection and severe inflammation, predominantly develops in the luteal phase [8]. A connection of bacterial infection with lipid accumulation cannot be excluded and it is supposed that lipids influence bacterial binding and adherence via scavenger receptors [3].

Lipid droplet accumulation in epithelial cells has formerly often been interpreted as a sign of degeneration. Meanwhile, it is clear that LDs are not only storage compartments for different classes of lipids in a cell, but they have also been identified as important dynamic cellular organelles and signal exchange platforms with key functions in lipid and energy homeostasis [9]. Therefore, investigating the lipid composition of the different endometrial tissues (connective tissue and epithelial tissue) is of high relevance for the interpretation of their function. In contrast to other cell organelles, LDs are surrounded by a phospholipid monolayer deriving from the endoplasmic reticulum (ER). On the outside, the phospholipid monolayer of the LDs is lined with various coating proteins such as perilipins (PLINs) which also support LD formation and stabilization. The family of PLINs in mammals actually comprises five members that are distinctive for different kinds of LDs (mainly concerning their cargo) with various functions [10]. Whereas PLIN1 is mainly restricted to differentiated adipocytes, PLIN2 (ADRP) and PLIN3 (TIP47) have been described in various tissues. PLIN2 is a marker of LD accumulation under physiological and pathological conditions [11]. It is present in a wide range of cells such as liver, skeletal muscle, macrophages, endotheliocytes, fibroblasts, adipocytes, myoblasts and also malignant cells [12–14]. PLIN1 and PLIN2 are also involved in the formation of LDs in steroidogenic cells [15]; these LDs might, beside other functions, provide a platform for active enzymes for steroidogenesis in addition to cholesterol ester storage. PLIN3 is necessary in the sorting of mannose 6-phosphate receptors in the Golgi apparatus [16, 17] and is expressed in almost all tissues, but especially in macrophages, atherosclerotic plaques, and hepatocytes [18]; its contribution to the biogenesis of LDs has been proven [19]. The presence and function of PLIN4 (S3–12) is quite unclear, it might be able to directly bind to neutral lipids [20]. It has also been reported to be present in heart and skeletal muscle [21] but it is absent in most other tissues except adipose tissue. PLIN5 (OXPAT, LSDP5) is an oxidative perilipin and has been detected in LDs of the myocardium and other oxidative tissues [22]. Due to their differential presence, PLINs might be useful tools to distinguish between LD species in the endometrium. Most LDs are present within the cytoplasm, however nuclear LDs were reported [23, 24] and should not be neglected in lipid investigations.

Our objective was to investigate the localization, quality and quantity of LDs in healthy and pyometra tissue samples to better understand their role the pathophysiology of pyometra in the dog. Futhermore, we assessed the expression of PLIN proteins (1, 2, and 3) on the protein and mRNA level to analyze their association with LDs. As we hypothesize that steroid hormones might be involved in the formation and accumulation of lipids in the canine endometrium, we conducted an experimental in vitro approach to study the potential of endometrial epithelial cells to response to external lipids along with steroid hormone stimulation to mimic sexual cycle conditions. This knowledge might help to elucidate the nature and function of LDs in the canine endometrium, which could be involved in both, physiological and pathophysiological processes.

## Material and methods

In brief, LDs were detected in uterine tissue samples from healthy and pyometra-affected bitches by means of histochemical methods (Oil Red O and/or p-phenylenediamine staining) and additionally characterized by the immunohistochemical detection of PLIN1, PLIN2, and PLIN3 as well as size distribution analysis using transmission electron microscopy. Canine endometrial epithelial cells in vitro models were used to further study the LDs biology under external lipid and hormone supplementation, focusing on LDs amount and interaction with lipid droplet coating proteins PLIN1–3.

### Tissue samples

All tissue samples were withdrawn from the archive of the VetBiobank/VetCore Facility for Research of the VetMedUni Vienna, Austria [25]. Archived tissues were obtained during routine ovariohysterectomy or therapeutic intervention according to the legal and ethical rules of the VetMedUni Vienna. Uterine tissue samples from healthy (*n* = 21; age from 9mo to 9y, documented body weight from 4.7 kg to 32,3 kg) and pyometra-affected bitches (*n* = 10; age from 1y 8mo 11y 8mo, documented body weight from 5.2 kg to 39 kg) of different breeds were used. Pyometra cases (4 open type, 2 closed type, 4 not stated) were determined as hyperplastic with severe inflammation, many/large cysts, increased endometrium/myometrium ratio and fibroblastic proliferation [26]. Samples were archived as fresh frozen pieces (shock frozen, stored in the gas phase over liquid nitrogen until use) for cryosections and additional as formaldehyde-fixed tissue pieces (4% neutral buffered formaldehyde for 24–48 hours) for paraffin embedded specimens (FFPE samples). The stage of the sexual cycle (luteal phase) was determined by morphological characteristics and analysis of the proliferation pattern as determined by Ki67 immunohistochemistry [27].

### Oil red O staining of tissue samples

Oil Red O (ORO) staining was applied on cryosections of healthy and pyometra affected endometrial cases for general lipid demonstration. Oil Red O stock solution was made by dissolving 0.5 g of ORO substance (Serva, Mannheim, Germany) in 100 ml of 2-propanol (Carl Roth GmbH, Karlsruhe, Germany). Working solution was prepared by diluting the stock solution with distilled water in a ratio of 6:4, 24 hours before the staining procedure. Cryosections were formaldehyde fixed (4% neutral buffered formaldehyde for 5 min), rinsed with distilled water, incubated in 60% 2-propanol (5 min at room temperature) followed by staining in ORO working solution (10 min at room temperature). Afterwards, samples were briefly rinsed in 60% 2-propanol (5-times, 2 sec each) and submerged into distilled water for 3 min. Cells were counterstained with hematoxylin solution for 3 min (Hematoxylin 1, Richard Allan Scientific, San Diego, CA, USA) followed by the blueing step in tap water. Finally, coverslips were mounted using Aquatex solution (Merck, Darmstadt, Germany).

For frozen tissue sections a semi-quantitative estimation score (0 = no staining, 1 = few LDs stained, 2 = moderate number of LDs stained, 3 = high number of LDs stained) was applied to evaluate the amount of lipid within the surface and glandular epithelium.

### Transmission Electron microscopy

Samples of healthy (*n* = 3) and pyometra affected canine endometria (*n* = 3) were fixed in 3% buffered glutaraldehyde (pH 7.4, Merck). After washing in 0.1 M phosphate buffer (Soerensen, pH 7.4) samples were postfixed in 1% osmium tetroxide (Electron Microscopy Sciences, Hatfield, PA, USA) for 2 hours at room temperature. Afterwards, samples were embedded in epoxy-resin (Serva). Semi-thin sections were cut at 0.8 μm, stained with toluidine blue (Merck) for general histological evaluation. Ultrathin sections were prepared (70 nm) and contrasted with uranyl acetate (Fluka Chemie AG, Buchs, Switzerland) and lead citrate (Merck), and evaluated by a transmission electron microscope (Zeiss EM900, Zeiss, Oberkochen, Germany). Images from surface and glandular epithelium were made to assess the amount of LDs (LDs/nuclei) and to measure LDs diameter (ImageSP-TRS, Moorenweis, Germany).

### P-phenylenediamine lipid staining of semithin sections

Semi-thin tissue sections (0.8 μm) of osmicated, resin-embedded tissue - as prepared for transmission electron microscopy (see above) - were used for lipid assessment with p-phenylenediamine (PPD). PPD staining is optimally applicable to identify lipids by light microscopy on semi-thin sections [28]. This staining allows distinguishing of lipids due to structural differences of the signal. P-phenylenediamine (Sigma Aldrich, St. Louis, MO, USA, 0.3 g) was dissolved in 30 ml ethanol. Semi-thin sections were stained for 5 min in the solution, washed by distilled water (2-times 2 min each) and mounted with Aquatex (Merck). Evaluation was performed using a bright-field microscope (Olympus BX53, Vienna, Austria).

### Immunohistochemistry

Ki67 immunostaining was used to confirm the luteal stage of the endometrial paraffin sections of healthy endometria. Immunostaining for PLINs (PLIN 1, 2, 3) was applied for LD analysis in both, healthy and pyometra-affected canine endometrial FFPE samples.

In brief, endogenous peroxidases were blocked in 3% H_2_O_2_ for 60 min, followed by a washing step in distilled water. Antigen retrieval was done by heating sections in 0.01 M citrate buffer, pH 6. Afterwards, sections were incubated in 10% normal goat serum (Sigma Aldrich) for 60 min to minimize unspecific binding, followed by the incubation with the primary antibody (antibodies and dilutions are summarized in Table 1) overnight at 4 °C. The next day, sections were incubated with the respective horseradish peroxidase (HRP) labelled secondary antibody (mouse or rabbit) and the signal detected by DAB (3′3’diaminobenzidine, Richard Allan Scientific, San Diego, CA, USA) reaction.

**Table 1:** List of antibodies used for immunohistochemistry and western blot detection

Antibody name	Method	Antibody typ	Source	Catalogue nr.	Dilution
**anti-PLIN1 (anti-PLIN1 rabbit polyclonal antibody)**	IHC	primary	Abcam	ab3526	1:10000
**anti-PLIN2 (ADRP (Perilipin 2) (AA5–27) mouse monoclonal antibody)**	IHC	primary	AntibodiesOnline	ABIN112185	1:1000
**anti-PLIN3 (TIP47 (F-10) mouse monoclonal antibody)**	IHC	primary	Santa Cruz	sc-390,968	1:5000
**anti-Ki-67 (Ki67 antigen clone MIB1 mouse monoclonal antibody)**	IHC	primary	Agilent Technologies	M7240	1:1000
**BrightVision, 1 step detection system goat anti-mouse HRP**	IHC	secondary	ImmunoLogic	DPVM110HRP	RTU
**BrightVision, 1 step detection system goat anti-rabbit HRP**	IHC	secondary	ImmunoLogic	DPVR110HRP	RTU
**anti-PLIN2 (ADRP/Perilipin 2 rabbit polyclonal antibody)**	WB	primary	Proteintech	15,294–1-AP	1:500
**anti-PLIN3 (TIP47 (F-10) mouse monoclonal antibody)**	WB	primary	Santa Cruz	sc-390,968	1:500
**anti-GAPDH (GAPDH [GT239] mouse monoclonal antibody)**	WB	primary	GeneTex	GTX627408	1:5000
**anti-α-tubulin (anti-α-tubulin rabbit polyclonal antibody)**	WB	primary	Abcam	ab4074	1:1000
**Amersham ECL rabbit IgG, HRP-linked whole AB (donkey)**	WB	secondary	GE Healthcare	NA934	1:5000
**Amersham ECL mouse IgG, HRP-linked whole Ab (sheep)**	WB	secondary	GE Healthcare	NA931	1:5000

*IHC* Immunohistochemistry, *WB* Western blot, *RTU* Ready to use

Immunostaining for PLIN2 and PLIN3 was evaluated by following estimation score: 0 = no staining, 1 = few LDs stained, 2 = moderate number of LDs stained, 3 = high number of LDs stained. Surface and glandular epithelium were scored separately. Due to the very contrasting distribution patterns of PLIN1, this scoring system was not applicable.

Positive and negative controls were carried along with each immunostaining. Negative controls included sections where the primary antibody was substituted by PBS to check for unspecific binding of the secondary system. Positive controls included sections of canine organs that are known to contain the protein of interest (adipose tissue for PLIN1, adrenal gland for PLIN2 and PLIN3, and intestine for Ki67).

### Western blot analysis

Western blots were applied to verify the specificity of the antibodies used for canine tissue. Frozen tissue samples were cut into small pieces, further disrupted and homogenised using a dounce homogenizer in RIPA lysis buffer (50 mM Tris-HCl pH 7.4, 500 mM NaCl 0.5% sodium deoxycholate (all Carl Roth GmbH), 1% Nonidet P-40 (Igepal, Sigma Aldrich/Merck, Darmstadt, Germany), 0.1% sodium dodecyl sulfate (Serva) supplemented with 1% (v/v) protease and phosphatase inhibitors (Protease Inhibitor Cocktail and Phosphatase Inhibitor Cocktail 3, both Sigma Aldrich). Lysates were then incubated on ice for 30 min and vortexed occasionally. To shred the DNA, lysates were pushed through a 20-G needle several times followed by centrifugation for 15 min at 4 °C using 10,621 x g. The soluble supernatant fraction was stored at − 80 °C until further analysis. Protein concentration was measured using DC™ Protein Assay (BioRad, Feldkirchen, Germany) according to the manufacturer’s recommendations. Protein extracts (20 μg protein/lane) were separated on 10% polyacrylamide minigels for SDS-PAGE and analyzed as described previously [29]. The primary and secondary antibodies are listed in Table 1. All antibodies were diluted in Western Blot Blocking Reagent (Roche, Mannheim, Germany)/TBST (1:10). For negative controls, the membranes were processed in the same way as described above, omitting the primary antibody. Positive controls were HepG2 cell lysate for PLIN2 and dog muscle tissue lysate for PLIN3.

### Cell culture and lipid supplementation experiments

Canine endometrial epithelial cells (cEECs) [30] were grown in Dulbecco’s modified Eagle’s medium containing 4.5 g/l glucose (DMEM, Sigma Aldrich) supplemented with 10% heat-inactivated fetal calf serum (FCS, Sigma Aldrich), 1% L-glutamine (Glutamine stable 100x, BioWest, Nuaille, France) and antibiotic-antimycotic solution (Antibiotic Antimycotic Solution Stabilized 100x, Sigma Aldrich) in a humidified atmosphere with 5% CO_2_ at 37 °C. Cells were regularly passaged twice a week using trypsin solution (Trypsin-EDTA, BioWest) with a usual split ratio 1:4.

For lipid and/or steroid hormone treatment, cells were seeded at a concentration of 4 × 10^4^ cells per well of a 24-well plate (TC-Platte 24 well standard F, Sarstedt, Nümbrecht, Germany) with or without glass coverslips (Cover glasses 12 mm, Menzel GmbH, Braunschweig, Germany). Alternatively, the same amount of cells was seeded per well of the 4-well glass chamberslide (Lab-Tek II Chamber Slide System, ThermoFisher Scientific, Waltham, MA, USA). The next day, treatment with oleic acid (Sigma Aldrich, final conc. 56.5 μg/ml), cholesterol (Sigma Aldrich, final conc. 50 μg/ml), or a combination thereof and solvent control ethanol (EtOH, Merck, final conc. 0.5%) was performed for 24 hours. For hormone treatment experiments, cells were cultured in the presence of progesterone (Progesterone water soluble, Sigma Aldrich, final conc. 30 ng/ml and 60 ng/ml), 17β-estradiol (17β-estradiol water soluble, Sigma Aldrich, final conc. 100 pg/ml and 200 pg/ml), oleic acid (Sigma Aldrich, final conc. 28.3 μg/ml) or a combination thereof for 24 hours. Afterwards, cells were washed in PBS and fixed with 4% neutral buffered formaldehyde (10 min at room temperature). After subsequent washing with water, cells grown on a glass coverslips were either air dried and stored for further histological analyses or subjected immediately to ORO staining. Cells grown in wells without coverslips were used for ORO staining followed by spectrophotometric quantification immediately. For quantitative analyses, data obtained from four independent experiments are shown.

### Oil red O staining of cells

To stain cultured cells in wells, fixed cells were incubated with 60% 2-propanol solution (250 μl/well for 5 min) followed by replacement with 250 μl of ORO (Serva) working solution for 10 min. After staining, wells were briefly washed first with 500 μl of 60% 2-propanol solution followed by water washing step. Staining of cells grown on glass coverslips was done as described above, doing all staining and washing steps in a glass cuvette. After staining, coverslips were mounted to the glass slides upside down using Aquatex (Merck).

### Quantification of Oil red O staining using image analysis

Image analysis for quantitative assessment of LDs in cultured canine endometrial cells after ORO staining was done with the open source software FIJI [31] and a self-designed macro plugin [32]. First, images made with a bright-field microscope (Olympus BX53, Vienna, Austria) were color deconvoluted with manually measured color vectors for ORO staining, hematoxylin (Hematoxylin 1, Richard Allan Scientific) counterstained nuclei and background to separate the different colors. The area of ORO staining was measured, using the same threshold for every image, which was primarily manually determined. The area was then related to the image size and given in percentage of the whole image area.

### Spectrophotometric quantification of the Oil red O staining

Further quantification of the ORO staining signal in canine endometrial epithelial cells was performed as described previously [33]. Briefly, stained cells in a 24-well plate were air dried, and the ORO stain was dissolved in 2-propanol (Sigma Aldrich, 500 μl/well, 15 min at room temperature). Afterwards, 200 μl of the respective 2-propanol sample was transferred per well of the 96-well plate (Greiner BioOne, Kremsmünster, Austria; each sample in duplicate) and the absorbance was read at 510 nm using Infinite M200 Pro Microplate Reader (Tecan, Grödig, Austria). Four independent biological replicates per group were prepared and used for further analysis unless otherwise indicated, while each absorbance measurement was performed in duplicates.

### Immunofluorescence

Canine endometrial epithelial cells were grown on either glass coverslips or chamber slides as described above, fixed in 4% neutral buffered formaldehyde for 10 min at room temperature, washed in distilled water and air-dried. For permeabilization, cells on coverslips/chamber slides were incubated in 0.2% TritonX-100 (Merck) in PBS for 15 min at 4 °C, followed by a washing step in PBS and incubation with the primary antibody overnight. Signal of the secondary HRP labelled antibody was detected using Invitrogen™ Alexa Fluor™ 488 Tyramide-Reagent (Invitrogen, Waltham, MA, USA) according to manufacturer’s guidelines, nuclei were counterstained using DAPI (4′,6-diamidino-2-phenylindole, Sigma Aldrich) and mounted with coverslip and Aqua Poly MountTM (Polysciences, Warrington, PA, USA).

Image analysis for quantitative assessment of PLIN2 immunostained LDs in cultured canine endometrial cells after ORO staining were done as described above (ORO staining) with the open source software FIJI and a self-designed macro plugin.

### Double labelling of Oil red O and PLIN2

As ORO is known to have a red fluorescence emission [34] double labelling in combination with immunofluorescence is possible. Canine endometrium epithelial cells (grown on chamber slides) were immunostained with PLIN2 antibody using the same protocol as mentioned including signal detection with Invitrogen™ Alexa Fluor™ 488 Tyramide-Reagent. Subsequently, ORO histochemical staining was performed as described above. At the end of the protocol, counterstaining of the nuclei was done using DAPI and preparations were mounted with Aqua Poly Mount™ and evaluated in a confocal microscope (Zeiss, LSM 880, Oberkochen, Germany).

### Quantitative real-time PCR

We analyzed the mRNA expression levels of genes involved in the fat metabolism encoding for fatty acid synthase (*FASN*), synaptosome-associated protein 23 (*SNAP23*), acetyl-CoA carboxylase 1 (*ACACA*), fatty acid binding protein 4 (*FABP4*) as well as genes for *PLIN1*, *PLIN2*, and *PLIN3*. Canine endometrial epithelial cells were lysed in TRI Reagent (Zymo Research, Irvine, CA, USA) and stored at − 80 °C until further processing. RNA extraction and DNase I treatment were done with the Direct-zol RNA Miniprep Kit (Zymo Research) according to the manufacturer’s instructions. Reverse transcription (RT) was performed with the High-Capacity cDNA Reverse Transcription Kit (Thermo Fisher). No-RT controls (without RT enzyme) were included for each sample to monitor the amplification of contaminating DNA. Primer for qPCR (Table 2) were designed with the PrimerQuest primer design tool (Integrated DNA Technologies, Coralville, IA, USA [35];), or taken from literature [3]. Quantitative PCR (qPCR) was done in 20 μl reaction volumes including 1x HOT FIREPol EvaGreen qPCR Mix Plus ROX (Solis BioDyne, Tartu, Estonia), 200 nM of each primer and 30 ng cDNA. All samples were analyzed in duplicates on a AriaMx Real-time PCR System (Agilent, Santa Clara, CA, USA) with following temperature profile: 95 °C for 12 min, 40 cycles of 95 °C for 15 s and 60 °C for 1 min, followed by a melting curve step (60 °C – 95 °C). Four potential reference genes (RGs): ornithine decarboxylase antizyme 1 (*OAZ1),* ribosomal protein L27 (*RPL27)*, ribosomal protein L32 *(RPL32)* and ribosomal protein L8 (*RPL8)* were included for normalization. The RG expression stability was assessed with the RefFinder tool [36] and the two most stably expressed genes *RPL27* and *RPL32* were selected for normalization. Target gene Cq values were normalized to the mean of the selected RGs and relative fold changes were calculated with the comparative 2-ΔΔCT method [37]. Shown are mRNA expression levels relative to those observed in cells cultured without any lipid supplementation.

**Table 2:** Primers used for RT-qPCR

Gene symbol	Gene name	NCBI accession number	Oligo sequence (5′ - 3′)	Amplicon length (bp)	PCR efficiency (%)	R^**2**^ value	Reference
***ACACA***	Acetyl-CoA carboxylase 1	XM_005624775.4, XM_005624776.4, XM_005624777.4, XM_038608922.1, XM_038608909.1, XM_005624778.4, XM_005624783.4, XM_548250.7	F: CGAGATTTCACTGTAGCTTCTCC R: TCATAAGACCATCGACGGATAGA	137	87	0.999	–
***FABP4***	Fatty acid binding protein 4	XM_038441472.1, XM_038516042.1, XM_038643854.1, XM_038579607.1, XM_845069.6	F: AGTTACTGCGGATGACAGAA R: ACGCCTTTCATGATACATTCC	150	105	0.998	–
***FASN***	Fatty acid synthase	XM_038675370.1, XM_038675367.1, XM_038675369.1	F: AGGAGTTCTGGGCCAATCTC R: GAGTGACGCTGGGTTGATG	242	93	0.998	–
***OAZ1***	Ornithine decarboxylase antizyme 1	NM_001127234.1	F: CTGCTGTAGTAACCTGGGTC R: ACATTCAGCCGATTATCAGAGTA	145	97	0.994	Gabriel et al., 2016
***PLIN1***	Perilipin 1	XM_038661340.1	F: GTACCCTCCTGAGAAGATTG R: GGGCACACTGATGCTATT	85	98	0.993	–
***PLIN2***	Perilipin 2	XM_005626663.3, XM_003639380.4	F: AATTTGCCAGAAAGAATGTGCAT R: TCCACCCAGGAGAGGTAGAACTT	79	99	0.991	
***PLIN3***	Perilipin 3	XM_038429023.1, XM_038429022.1	F: GGGTCAGGAGAAACTACAC R: GTCTCCACCTCTGGTTTG	93	97	0.995	–
***RPL27***	Ribosomal protein L27	NM_001003102.2	F: ACTACAATCACCTCATGCCC R: CTTGTACCTCTCCTCGAACTTG	143	94	0.998	Gabriel et al., 2016
***RPL32***	Ribosomal protein L32	NM_001252169.1	F: TGGCCATCAGAGTCACCAATC R: GACGCGCACATAAGCTGTTTAT	74	94	0.998	–
***RPL8***	Ribosomal protein L8	XM_853403.4	F: TCTTCCGCCAACAGAGCC R: CTTTGCCTTGTACTTGTGGTAAGC	102	94	0.995	–
***SNAP23***	Synaptosome-associated protein 23	XM_038442089.1, XM_038442090.1, XM_038442091.1, XM_038442087.1, XM_038442088.1	F: TGGATAATCTGTCACCAGAAGAAA R: TCCTGAGACTCAATGGCTAAAC	106	90	0.999	–

R^2^: correlation coefficient of standard curve; F and R: forward and reverse primer

### Statistical analysis

Statistical analyses (unpaired t-test with Welch’s correction) were conducted with GraphPad Prism 8.4.3 (GraphPad Software, San Diego, CA, USA). A *p*-value < 0.05 was considered as statistically significant.

## Results

### Tissue sections

**Fig. 1:**
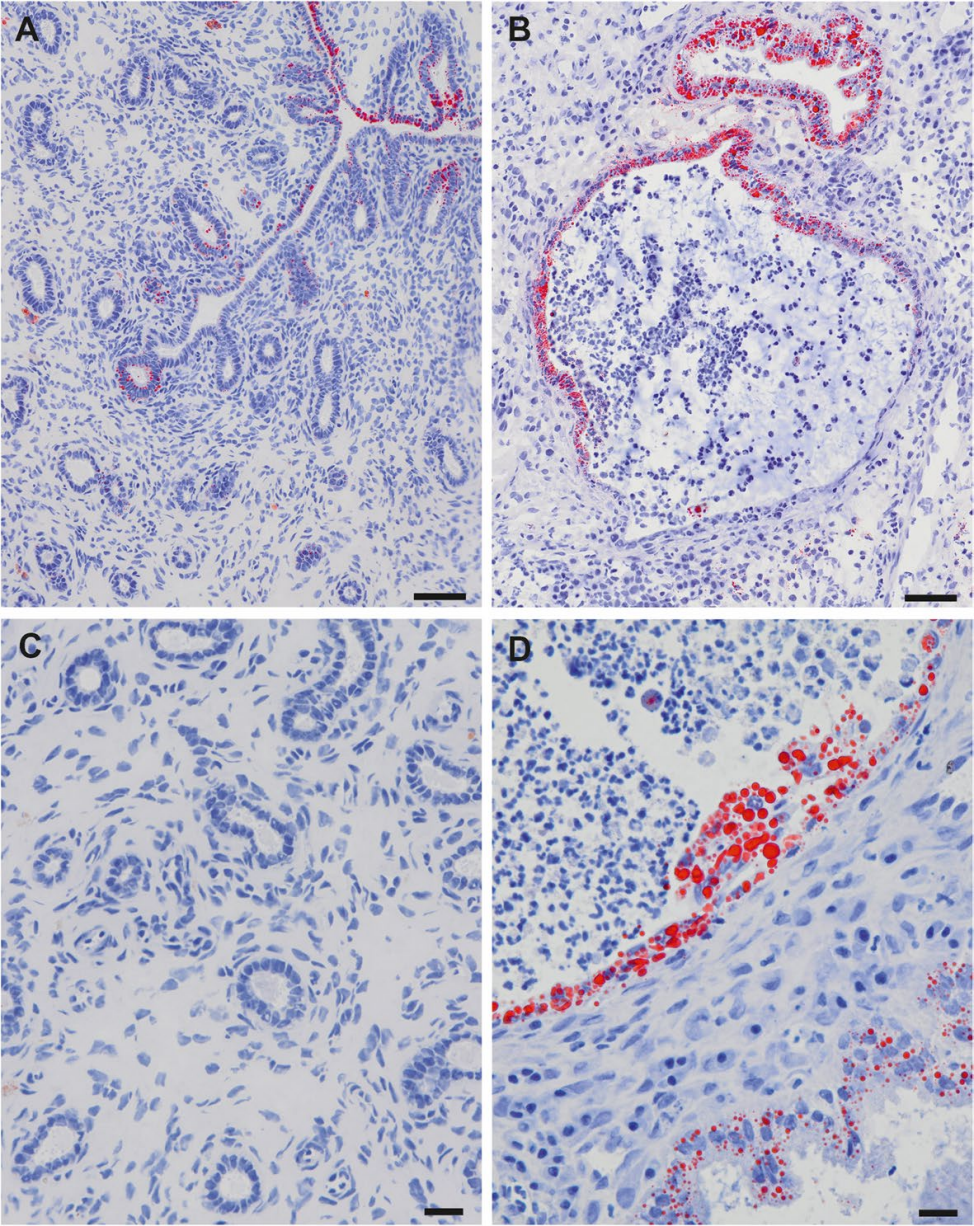
ORO staining of cryosections of healthy (**A**, **C**) and pyometra affected (**B**, **D**) canine endometrium. Positive signals were observed in the surface epithelium of the healthy and diseased endometrium. No lipid droplets were detected by ORO staining in the uterine glands of healthy samples (**C**); however, strong signals were present in dilated glands and crypts in pyometra sections. Scale bars (**A**, **B**) = 50 μm, (**C**, **D**) = 20 μm

The sexual cycle stage of healthy uterine tissue sections was determined by Ki67 immunostaining pattern. As characteristic for luteal stage, proliferative activity was generally low; Ki67 immunohistochemistry resulted in a characteristic staining pattern with only scattered positive cells in the surface epithelium, superficial glands, and stroma. Ki67 immunostaining was absent in basal glands. All pyometra samples had a high proliferative activity within the stroma (inflammatory cells) and scattered positive cells in the surface and crypt epithelium (not shown). To visualize the general amount of LDs in the healthy and diseased endometrium, we applied ORO histochemistry on frozen tissue sections. Scoring of ORO staining in tissue sections of healthy canine endometrium on average revealed a moderate amount of LDs in the surface epithelium whereas the glandular epithelium was almost free of LDs (Fig. 1). Pyometra samples had a higher number of LDs in epithelial tissues compared to the healthy endometrium (Fig. 1). Total scoring results of ORO staining are summarized in Table 3.

**Table 3:** Oil Red O staining in epithelial compartments of the endometrium

Oil Red O	No staining (0) %	Low (1) %	Moderate (2) %	Strong (3) %
	healthy/pyometra	healthy/pyometra	healthy/pyometra	healthy/pyometra
**Surface epithelium**	0/0	35/0	40/11	25/89
**Glandular epithelium**	90/43	5/14	5/43	0/0

Immunohistochemistry for different members of the PLIN family (PLIN1, PLIN2, and PLIN3) intended to specify the LD coating and structure in the canine endometrium. Although all PLIN proteins are somehow involved in LD formation and/or LD coating, their distribution in the canine endometrium varied greatly. PLIN1 was negative in all endometrial structures, but stained lipid vacuoles in unilocular fat cells that were present occasionally in the perimetrium (not shown). Therefore, quantification as initially intended for the epithelial endometrial cells was not possible for PLIN1.

**Fig. 2:**
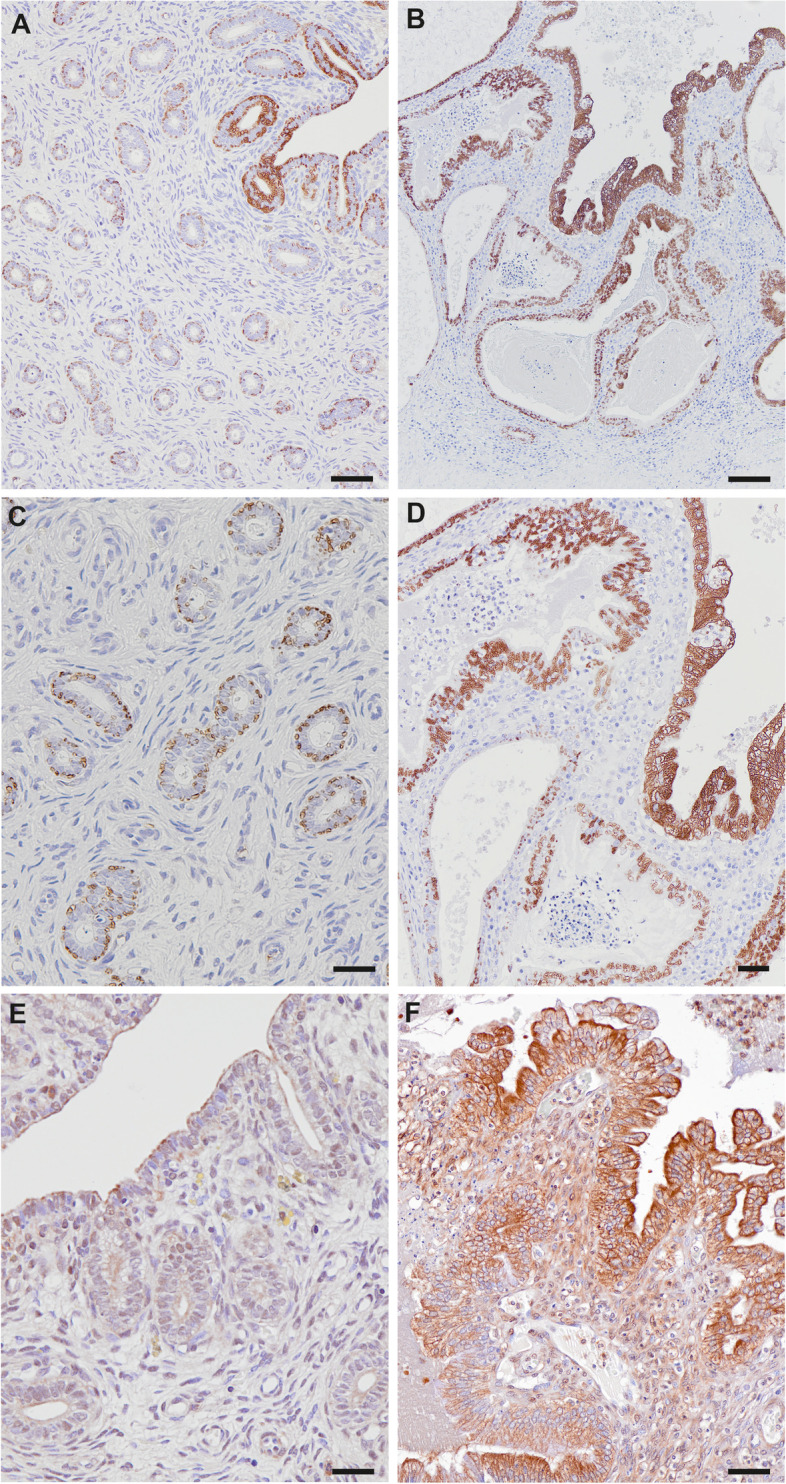
Immunohistochemistry for PLIN2 (**A**-**D**) and PLIN3 (**E**, **F**) on paraffin sections. In the healthy endometrium PLIN2 positive lipid droplets were present in the surface (**A**) and glandular epithelium (**C**). In pyometra samples a high number of PLIN2 stained lipid droplets were seen in surface, glandular and crypt epithelia (**B**, **D**). Healthy endometrial samples were almost devoid of PLIN3 signals (**E**), whereas pyometra sections showed moderate to strong signals in the surface epithelium (**F**). Scale bars (**A**, **B**) = 100 μm, (**C**) = 20 μm, (**D**) = 50 μm, (**E**, **F**) = 50 μm

PLIN2 immunostaining clearly marked the membrane of LDs. Amount and distribution of PLIN2-positive LDs in the surface epithelium was comparable with ORO staining. In contrast, a high number of PLIN2 positive LDs was also observed in the endometrial glandular epithelium, whereas ORO staining was absent in most endometrial glands. In pyometra specimens, the amount of PLIN2-positive LDs was generally higher compared to healthy samples (Fig. 2, Table 4). PLIN3 was strongly expressed in the cytoplasm but not clearly associated to LDs. PLIN3 expression was also higher in pyometra samples compared to healthy endometrium (Fig. 2, Table 5).

**Table 4:** Immunohistochemical detection of PLIN2 in epithelial compartments of the endometrium

PLIN2	No staining (0) %	Low staining (1) %	Moderate (2) %	Strong (3) %
	healthy/pyometra	healthy/pyometra	healthy/pyometra	healthy/pyometra
**Surface epithelium**	14/0	24/0	14/30	48/70
**Glandular epithelium**	19/0	38/0	24/60	19/40

**Table 5:** Immunohistochemical detection of PLIN3 in epithelial compartments of the endometrium

PLIN3	No staining (0) %	Low staining (1) %	Moderate (2) %	Strong (3) %
	healthy/pyometra	healthy/pyometra	healthy/pyometra	healthy/pyometra
**Surface epithelium**	67/0	19/0	10/10	4/90
**Glandular epithelium**	67/30	10/10	19/30	4/30

Western blot detection of PLIN2 and PLIN3 protein was performed in whole tissue extracts prepared from healthy and diseased canine uteri. Specific bands for PLIN2 (50 kDa) and PLIN3 (40 kDa) were observed with different intensities among the tested samples as well as in corresponding positive controls.

**Fig. 3:**
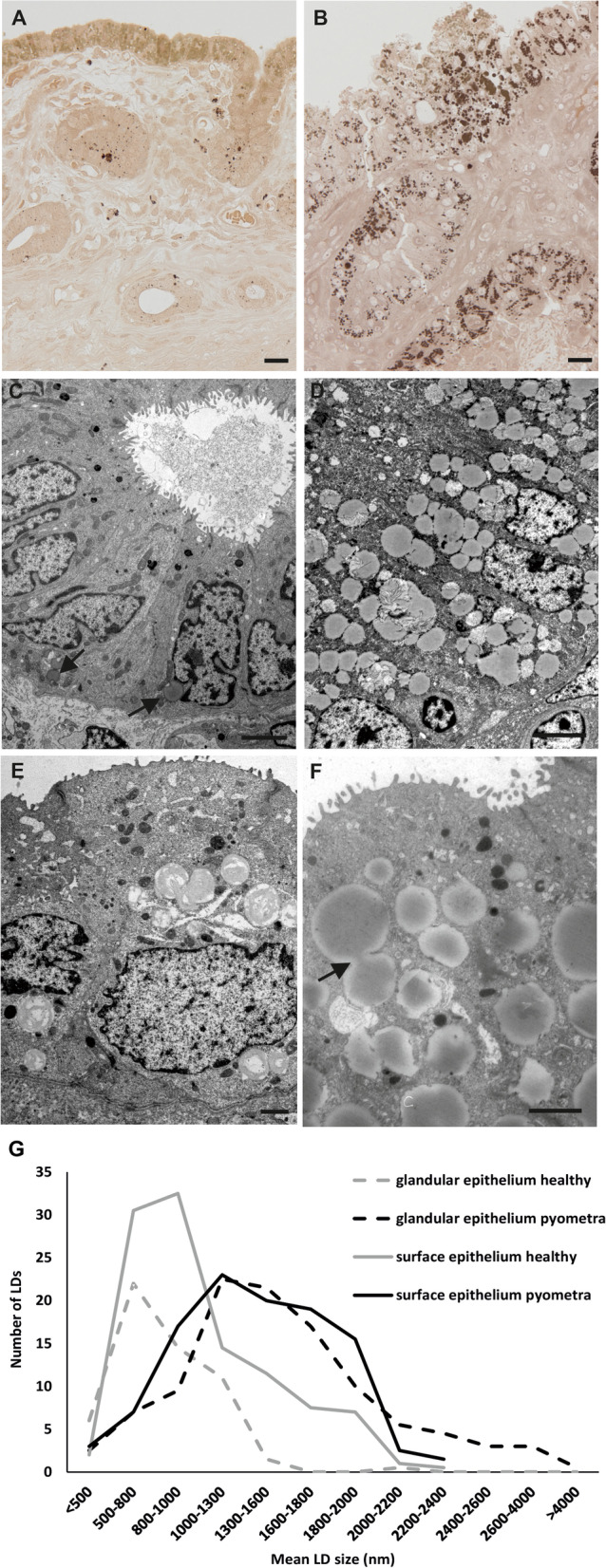
Healthy (**A**) and pyometra (**B**) resin semi-thin sections stained with PPD. Light brown stained lipid droplets are seen in the surface epithelium of the normal endometrium. In contrast, dark stained lipid droplets were found in the glandular structures (**A**). Lipid droplets in pyometra samples showed predominantly dark staining in all endometrial epithelia (**B**). TEM micrographs of healthy glandular (**C**) and surface (**E**) endometrial epithelium. Only a limited number of lipid droplets was seen in the glands (arrows). Pyometra sections of surface epithelium (**D**, **F**) had a higher incidence of lipid droplets and lipid droplets were larger. Fusion of lipid droplets was frequently observed in pyometra samples (arrow) (**F**). Note, that the lipid droplets differ in their inner structures. Scale bars (**A**, **B**) = 20 μm, (C, D, F) = 2500 nm, (**E**) = 1000 nm. (**G**) Results of measurement of lipid droplets in TEM ultrathin sections. LD size was increased in pyometra surface and glandular epithelium compared to healthy surface and glandular epithelium

PPD staining of resin semi-thin sections showed distinguishable LDs as marked by different dyeing. Large, light brown, homogenous LDs were observed in the surface epithelium of healthy samples, whereas only scattered tiny, dark stained LDs were seen in the glands. LDs in pyometra samples were generally larger and stained darker compared to LDs in healthy samples (Fig. 3). Transmission electron microscopy allowed to precisely determine the size of LDs and to estimate LD number in healthy and diseased canine endometrial epithelia. Specimens had high individual variability with regards to number and size of LDs, however, a clear increase of LD size was observed in pyometra samples in surface and glandular epithelium compared to healthy samples (Fig. 3A, B, G). Fusion of LDs was regularly observed in pyometra samples. Transmission electron microscopy also revealed differences in the ultrastructure of LD content. Some LDs showed a lamellar appearance whereas others were characterized by a homogenous content (Fig. 3).

### Cell culture

**Fig. 4:**
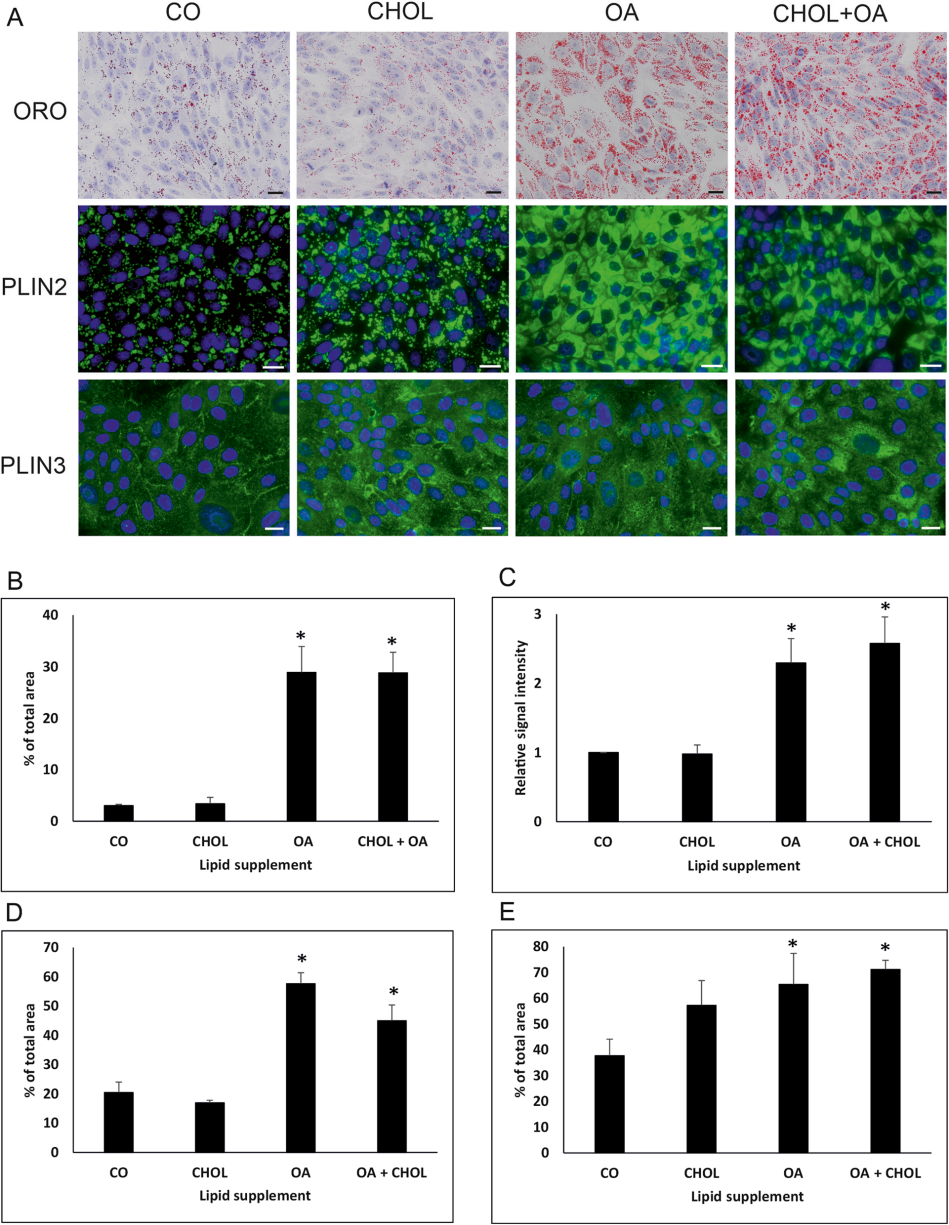
Qualitative (**A**) and quantitative (**B**, **C**, **D**, **E**) analysis of ORO staining and PLIN2/PLIN3 expression in canine endometrial epithelial cells treated with lipid supplements. Cultured cells were treated with cholesterol (CHOL) or oleic acid (OA) or both (CHOL+OA). Staining with ORO and immunohistochemistry for PLIN2 revealed an increase of lipid droplets after OA and CHOL+OA treatment. Scale bars = 20 μm. Imaging analysis of ORO (**B**), PLIN2 (**D**), and PLIN3 (**E**) immunohistochemistry resulted in a statistically significant increase of the measured parameters in both OA- and OA + CHOL-treated samples. Similarly, quantitative spectrophotometric assessment of ORO staining (**C**) revealed a significant increase of signal intensity in cells treated with OA alone or in combination. * *p*-value versus untreated control (CO) < 0.05

ORO staining was used to qualitatively and quantitatively assess LDs in canine endometrial epithelial cells grown in culture medium with and without lipid supplementation to determine processing of external lipids by these cells. Cells grown without lipid supplementation (not treated and ethanol diluent supplemented groups) revealed only a limited and comparable amount of LDs of relatively homogeneous size, distributed irregularly within the cell. However, a significant rise of LDs was observed in cells treated with oleic acid alone or in combination with cholesterol (Fig. 4A). Cell cultivation in medium supplemented with cholesterol alone did not have an impact on LDs, keeping their amount similar to those observed in control cells (without supplementation).

To quantify the intensity of the ORO staining signal, image analysis as well as spectrophotometric quantification was performed. Using image analysis, quantitative data represent the percentage of area of ORO signal calculated out of the whole area covered with cells. A significant rise in positive signals was observed when comparing not treated groups or ethanol diluent supplemented groups to cells supplemented with oleic acid. Supplementation with a combination of both oleic acid and cholesterol did not yield a further increase in signal, whereas supplementation with cholesterol alone had no effect (Fig. 4B). Similar to previous observation(s), a significant increase (2–3-fold) in relative signal intensity was also observed in samples treated with oleic acid alone or in combination with cholesterol when compared to control cells as determined by spectrophotometric quantification (Fig. 4C). PLIN2 immunostaining of canine endometrial epithelial cells resulted in a pattern comparative to ORO with a significant increase of LDs by oleic acid or oleic acid and cholesterol treatment but not with cholesterol alone (Fig. 4A, D). PLIN3 immunostaining revealed a granular cytoplasmic signal in canine endometrial epithelial cells. Due on the appearance of large LDs in the cytoplasm of cells after oleic acid treatment, its distribution pattern was changed moderately (Fig. 4A). Again, a statistically significant rise in positive signals was observed in groups treated with oleic acid alone and oleic acid plus cholesterol compared to non-treated control group (Fig. 4E).

**Fig. 5:**
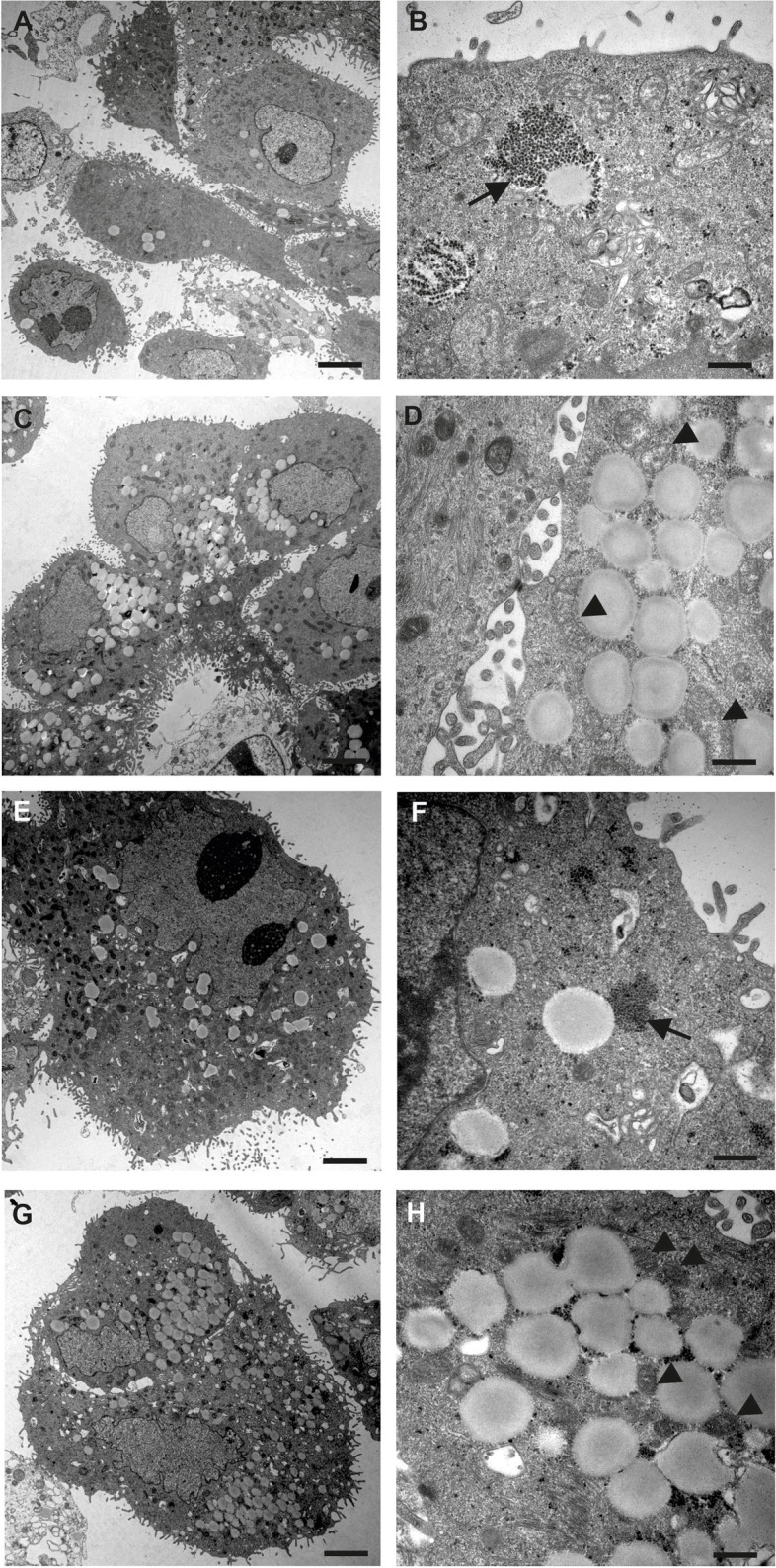
Multiplex fluorescent labelling of cultured canine endometrial epithelial with PLIN2 (green), ORO (red) and DAPI (magenta). Cells treated with oleic acid (OA; **A**, **B**) and cells cultured in normal medium (CO; **C**) showed only partial co-localization of ORO and PLIN2 signals in LDs. LDs with ORO and PLIN2 signal (arrow), LDs with only PLIN2 signal (arrowheads), LDs with only ORO signal (circle). Scale bars (**A**) = 20 μm, (**B**, **C**, **D**) = 5 μm

Due to the partly differential PLIN2 and ORO staining patterns in tissue sections and cell cultures, we applied a double labelling method combining immunofluorescence for PLIN2 and histochemical ORO staining to elucidate if both markers are concomitantly present on/in LDs in vitro. Multiplex-labelled cell cultures of canine endometrial epithelial cells revealed the presence of ORO and PLIN2 single positive droplet-like structures as well as co-labelled LDs independent of treatment (Fig. 5).

**Fig. 6:**
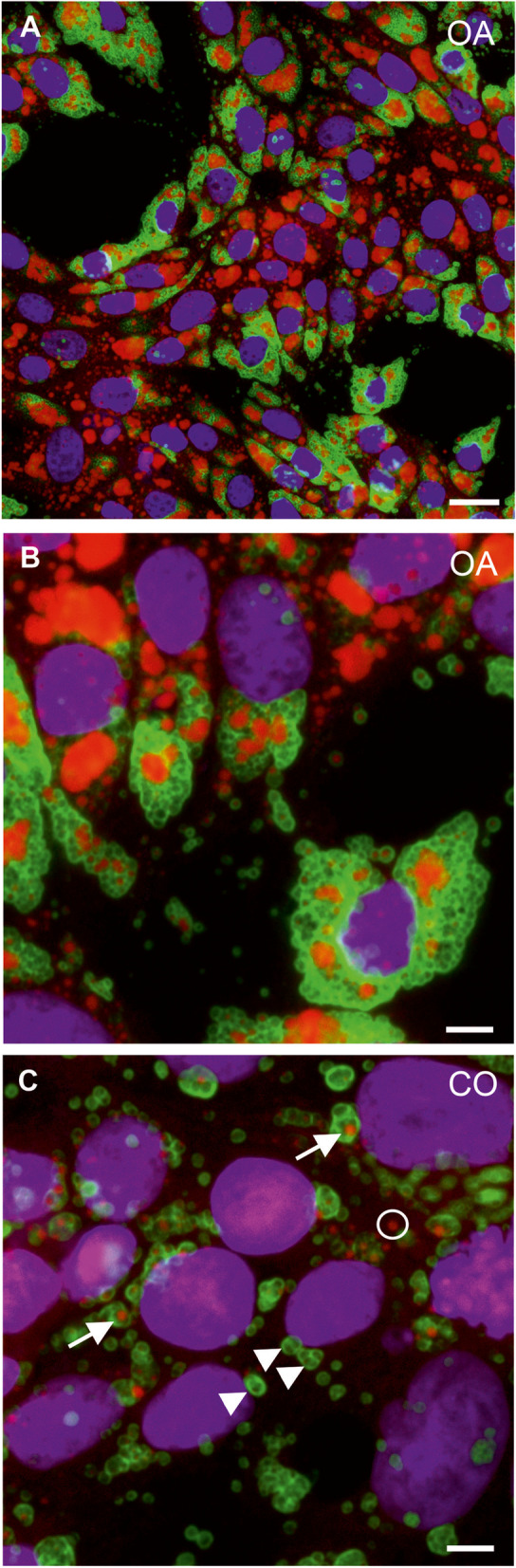
TEM of cell cultures of canine endometrial epithelial cells in normal medium (**A**, **B**) and after treatment with oleic acid (**C**, **D**), cholesterol (**E**, **F**) or oleic acid and cholesterol (**G**, **H**). There is a significant increase of LDs in the cells if stimulated by external oleic acid alone or in combination with cholesterol. Lipid droplets were often situated adjacent to glycogen aggregations (arrow) or mitochondria (arrowhead) independently of experimental group. Scale bars (**A**, **C**, **E**, **G**) = 2500 nm, (B, D, F, H) = 500 nm

Transmission electron microscopy of cultured canine endometrial epithelial cells revealed a moderate number of LDs in untreated cells, after supplementation with oleic acid (56.5 μg/ml) or/and cholesterol (50 μg/ml) the number of LD increased significantly. LDs were frequently framed by glycogen (Fig. 6).

**Fig. 7:**
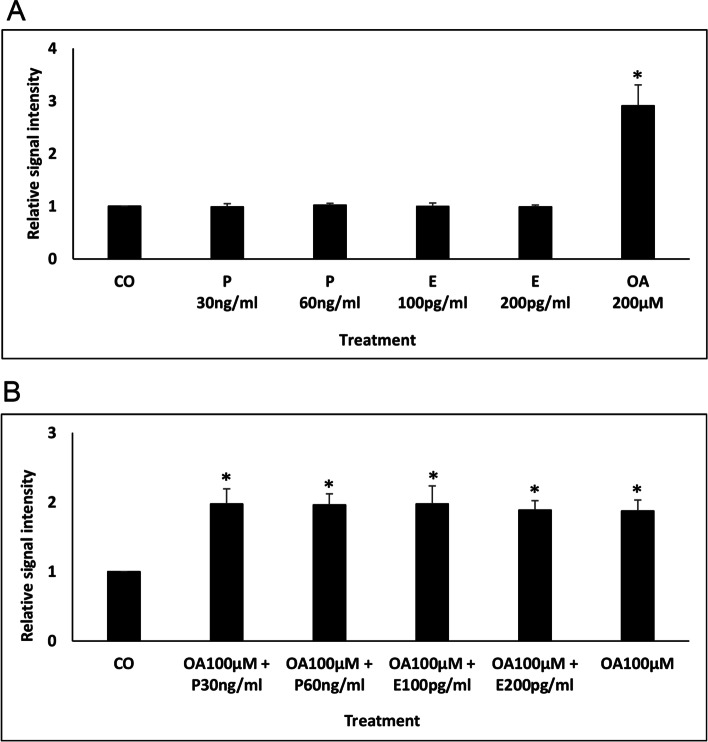
Quantitative analysis of ORO staining in canine endometrial cells after stimulation with 17ß-estradiol or progesterone alone (**A**) or in combination with oleic acid (**B**). Hormone stimulation alone did not affect the amount of LDs as measured by ORO spectrophotometric assay (**A**). Stimulation with oleic acid (OA) concomitantly with hormonal treatment did not show an enhancing/additional effect beside the significant increase of LDs independent of steroid hormone presence (**B**). P - progesterone, E - 17ß-estradiol. * *p*-value versus untreated control (CO) < 0.05

As LDs in the canine endometrium are clearly associated with certain stages of the sexual cycle, hormonal governance on their formation, and subsequently amount and size of LDs is supposed. We stimulated canine endometrial epithelial cells in vitro with 17ß-estradiol and progesterone to find out if these steroid hormones influence their lipid metabolism and thereby LD number. Stimulation of the cells with the respective hormone in different concentrations did not change the amount of LDs as measured by ORO spectrophotometric assay (Fig. 7A). On top stimulation with oleic acid (final concentration 28.3 μg/ml) concomitantly with hormonal treatment showed no extra effect beside the reaction already demonstrated by previous experiments (significant increase of LDs independent of steroid hormone presence; Fig. 7B).

**Fig. 8:**
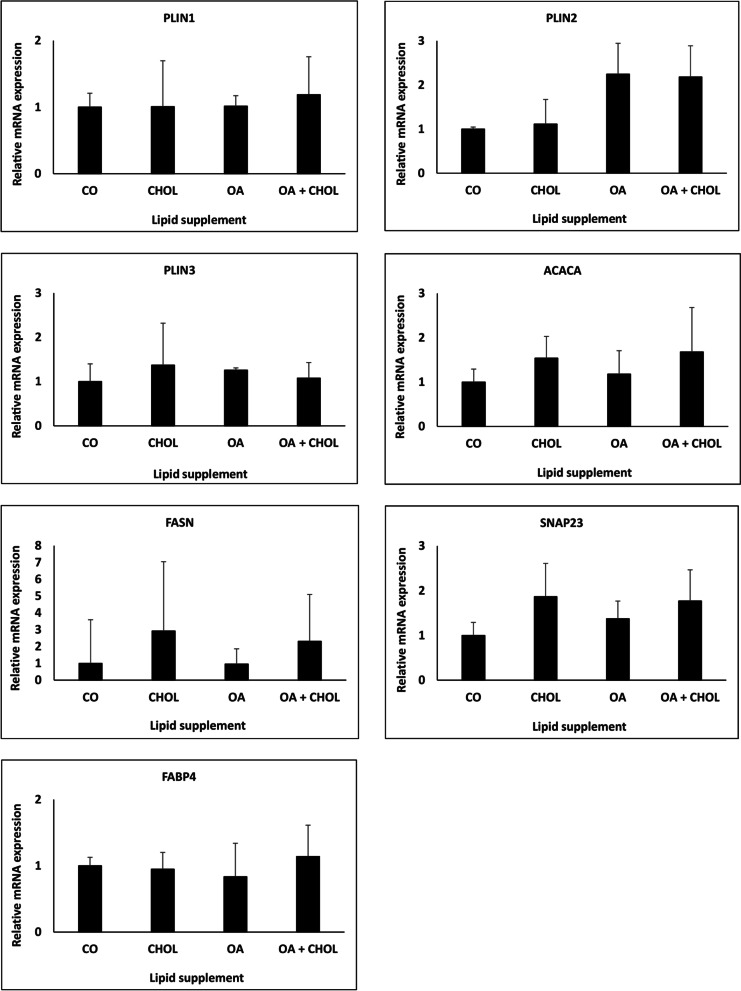
Effect of lipid supplements on mRNA expression of selected genes involved in lipid droplet formation and lipid metabolism. Relative expression levels of mRNA encoding for *PLIN1*, *PLIN2*, *PLIN3*, *ACACA*, *FASN*, *SNAP23*, and *FABP4* were quantified using RT-qPCR. * *p*-value versus untreated control (CO) < 0.05

Last but not least, the effect of lipid supplementation on mRNA expression levels of selected proteins involved in LDs formation - *PLIN1, PLIN2, PLIN3* and lipid metabolism - *FABP4*, *ACACA*, *FASN*, and *SNAP23* was studied in the canine endometrial epithelial cells in vitro. No statistically significant changes in mRNA expression levels were observed between cells grown with or without lipid supplementation (Fig. 8; Supplementary Fig. 1).

## Discussion

The current investigation intended to determine amount, localization and composition of LDs in canine endometrial cells and thereby contribute to elucidate their function in this organ. Listed potential functions of LDs are manifold and include, besides being energy reservoirs, precursors for steroidogenesis (cholesterol), providing fatty acids for prostaglandins, protection against lipotoxicity, facilitating coordination and communication between intracellular organelles, and many others (for review see 9, [38]). LDs in the endometrium have already been described in several species, including rat, cow, and sheep [6, 7, 39]. During luteal phase, the epithelium is highly active in secretion and beside that, LDs are also present in a substantial amount [3, 40]. The reason for this cyclic lipid storage in the canine endometrium has not been uncovered so far, and the lipid composition accumulated within the LDs and their coating proteins are still unknown.

The incidence of lipids in the endometrium – especially in conjunction with the development of a corpus luteum - has early been observed [4]. Their presence has mostly been interpreted as an essential part of the histotrophe in the pregnant uterus [41]. This hypothesis is supported by the discovery of lipids, namely fatty acids and oxylipins, accumulating in the uterine lumen during dioestrus [39]. In their review, Ribeiro et al. [42] stated that endometrial lipids are the most important source of fatty acids for utilization by the conceptus and become available in the uterine lumen through exporting of exosomes, microvesicles, carrier proteins and lipoproteins. In the canine endometrium, this function must also be considered as the maternal placenta (glandular chambers) has a conspicuous presence of LDs in the dog.

The aim of the present study was to characterize the LDs in the epithelial cells of the canine endometrium by classical histochemical lipid staining (ORO) but also by means of immunohistochemistry, identifying LD coating proteins (PLINs), as these have been found to be predominantly associated with LDs in certain cell types (reviewed in [38]). ORO staining and transmission electron microscopy were performed to determine the number and size of LDs in the canine endometrium, thereby demonstrating that mean number and size of LDs were increased in pyometra samples compared to healthy samples. Based on that longer presence, LDs have a tendency to fuse, which would explain their larger size as observed in TEM. The fusion of LDs in mammalian cells leading to their size increase has been verified and is mediated by the cell death-inducing DNA fragmentation factor alpha (DFFA)-like effector (CIDE) family of proteins [9, 43]. Fusion events of LDs have been frequently observed during our transmission electron microscopy analysis in canine endometrial epithelial cells. Markers for fusion processes like the ones mentioned above, should be included in future investigations to verify this hypothesis although it might be difficult to obtain validated dog-specific antibodies.

In the present study members of the mammalian PLIN family were assessed by immunohistochemistry in healthy and pyometra affected tissue sections. Ki67 immunostaining patterns suggestl that in canine pyometra affected endometrium the characteristic cyclic patterns are lost, as has been described before [44]; this has to be considered during interpreting LDs. PLIN2 and PLIN3 were abundantly present in the epithelia of the canine endometrium. While PLIN2 was strictly associated with LDs, we found PLIN3 not always clearly correlated to LDs. Data about PLIN2 in the endometrium, even in humans, are rare. It is known, that PLIN2 directly interacts with lipids on the surface of lipid droplets and influences levels of key enzymes and lipids involved in maintaining LD structure and function [45]. In addition, PLIN2 prevents human trophoblast from an apoptotic cell death [46]. The data situation about PLIN3 is even worse. To our best knowledge, no studies about PLIN3 in the endometrium have been carried out. In the wider context of reproduction, PLIN3 (aliases TIP47, PP17) was found to be associated to LDs in placenta [47]. Its functions, beside mannose-6-phosphate receptor transportation from the endosome to the Golgi-apparatus, are still debated. It seems clear that it is involved in the biogenesis of LDs [19]. In our analyses, PLIN2 and PLIN3 were more abundant in pyometra-affected endometrium samples compared to healthy ones. Therefore, the amount and distribution of PLIN2 associated with LDs or PLIN3 in the cytoplasm might indeed be connected to the disease.

To enable the investigation of the responsiveness of canine endometrial cells to external lipid sources and thereby the dynamics of LD formation, we made an experimental in vitro approach by using primary canine endometrial cells [30]. In line with our observations in endometrial epithelial tissue, cell cultures of canine endometrial epithelial cells revealed presence of LDs as demonstrated by ORO staining as well as positive PLIN2 immunostaining. Stimulation with oleic acid led to a significant increase of LDs in cultured endometrial epithelial cells in contrast to cholesterol stimulation, which had no effect. LDs in canine endometrial epithelial cells in vitro were positive for ORO and PLIN2. Therefore, we suggest that LDs in the canine endometrium are predominantly composed of neutral lipids and not cholesterol, which supports the hypothesis of LDs being an energy source for the conceptus. However, other functions are possible, as they could also provide fatty acids for prostaglandin synthesis. Interestingly, although the general trend of staining pattern and intensity was identical between ORO and PLIN2, the individual pattern of positive LDs (as marked by ORO) was not identical to the PLIN2 pattern. This discrepancy suggests that PLIN2, although being the most frequent LD-associated protein, is missing on a cohort of lipid droplets. It might also indicate a difference in the content of LDs in the examined cells. This hypothesis was further supported by the results obtained by means of transmission electron microscopy, in vitro experiments, and double fluorescence labelling of ORO and PLIN2. It has to be considered that ORO does not stain all species of lipids such as unesterified cholesterol [48].

As LD accumulation peaks in luteal phase, we assessed a potential hormonal regulation of the lipid processing. Other authors [7, 39, 49] stated that endometrial LD expression might be regulated by steroid hormones and other factors controlling reproductive cyclicity, and regulation of LD formation within the endometrium may be species-specific. However, in our in vitro experiments, stimulation with 17ß-estradiol or progesterone had no significant influence on LD formation and number in canine endometrial epithelial cells. Admittedly, this experiment was performed on only one primary cell line so far and should be extended to distinct canine endometrial epithelial cell lines. The loss of cycle-specific proliferation patterns in pyometra samples, and therefore cellular characteristics, might also be reflected by presence and morphology of LDs.

Next to the detection of LD associated proteins we investigated mRNA levels of several genes involved in LD formation and lipid metabolism. Fatty acid synthase (*FASN*) mRNA was previously detected in the human endometrium [50]. Its mRNA levels were significantly higher in endometrial epithelial cells than in fibroblasts. In both cell types, mean *FASN* mRNA concentrations were higher in biopsies removed during the luteal phase than the follicular phase of the menstrual cycle [50]. Enhanced *FASN* mRNA levels were observed in white adipose tissue after treatment with trilactic glyceride [51]. In our experiments, lipid supplementation did not show any significant effect on *FASN* mRNA levels in canine endometrial epithelial cells. The synaptosomal-associated protein 23 (*SNAP23*) is involved in LD formation and fusion as mentioned above [52–54] as well as in the interaction between LDs and mitochondria [55]. Our data revealed an increase in LD formation in canine endometrial epithelial cells treated with oleic acid and contacts between LDs and mitochondria were frequently observed in canine endometrial cells in vitro by transmission electron microscopy. Therefore, we expected an increase in *SNAP23* mRNA levels in treated cells. Surprisingly, similar as for *FASN*, no significant changes for *SNAP23* were observed among the tested samples. The fatty acid binding protein 4 (*FABP4*) was found to be expressed by epithelial cells of the proliferative endometrium, as well as epithelial and stromal cells of secretory endometrium [56]. *FABP4* has an important role in the establishment and maintenance of pregnancy, with decreased expression of the protein in the endometrium possibly linked to pregnancy loss [57]. *FABP4* levels were remarkably increased under high ω-3 PUFA exposure [58], however our treatment with lipid supplements revealed no effect on the mRNA level. To our best knowledge, no relevant data describing levels of acetyl-CoA carboxylase-alpha (*ACACA*) in uterine tissue or cells have been presented up to the publication date of the current study. However, treatment of primary hepatocytes with cholesterol increased the mRNA level of *ACACA* and *FASN* [59]. This is again in contrast to obtained results in canine endometrial epithelial cells. Thus, our hypothesis, that genes which are associated markers in the lipid metabolism in cells are regulated in correlation with LD formation has been disproven. Expression levels of the selected genes of interest (*FASN, SNAP23, ACACA, FABP4*) were not changed after external lipid stimulation of canine endometrial epithelial cells.

In the frame of this investigation we intended to promote the analysis of LDs in the canine endometrium as they have not gained much attention until now. However, due to their vast presence in the uterus, specific functions during the reproductive process are likely. We found differences in the healthy and pyometra-affected endometrium and could show the responsiveness of endometrial epithelial cells to external lipids. Moreover, several kinds of LDs seem to be present in the canine endometrium. In conclusion, LDs were found in endometrial surface and glandular epithelium in both healthy and pyometra-affected bitches. We assume that LDs in the canine uterus mainly contains triglycerides and therefore in general provide energy for the developing embryo. A clear increase in LD size was observed in samples from pyometra-affected bitches, in both evaluated epithelial regions. Therefore, we speculate that LDs might be involved in the pathophysiological processes of pyometra. Limiting factors of our study are the relative low number of cases and the restriction of one region of the uterus which was due to availability of fresh frozen material. Nevertheless, we hope that with this study we created a focal point that encourages further groups to join the lipid research in reproduction.

## Supplementary Information

**Additional file 1.**

## Data Availability

All data generated or analysed during this study are included in this published article and its supplementary information files. Datasets used and/or analysed during the current study are available from the corresponding author on reasonable request.
